# Mitigating Increased Cardiovascular Risk in Patients with Obstructive Sleep Apnea Using GLP-1 Receptor Agonists and SGLT2 Inhibitors: Hype or Hope?

**DOI:** 10.3390/biomedicines12112503

**Published:** 2024-11-01

**Authors:** Paschalis Karakasis, Marios Sagris, Dimitrios Patoulias, Theocharis Koufakis, Panagiotis Theofilis, Aleksandra Klisic, Nikolaos Fragakis, Mohamed El Tanani, Manfredi Rizzo

**Affiliations:** 1 Second Department of Cardiology, Hippokration General Hospital, Aristotle University of Thessaloniki, 54642 Thessaloniki, Greece; fragakis.nikos@googlemail.com; 2 First Cardiology Department, School of Medicine, Hippokration General Hospital, National and Kapodistrian University of Athens, 15772 Athens, Greece; masagris1919@gmail.com (M.S.); panos.theofilis@hotmail.com (P.T.); 3 Second Propedeutic Department of Internal Medicine, Faculty of Medicine, School of Health Sciences Aristotle, University of Thessaloniki, 54642 Thessaloniki, Greece; dipatoulias@gmail.com (D.P.); thkoyfak@auth.gr (T.K.); 4 Primary Health Care Center, Faculty of Medicine, University of Montenegro, 81000 Podgorica, Montenegro; aleksandranklisic@gmail.com; 5 Ras Al Khaimah Medical and Health Sciences University, Ras Al Khaimah P.O. Box 11172, United Arab Emirates; eltanani@rakmhsu.ac.ae (M.E.T.); manfredi.rizzo@unipa.it (M.R.); 6 School of Medicine, Department of Health Promotion, Mother and Child Care, Internal Medicine and Medical Specialties (Promise), University of Palermo, 90100 Palermo, Italy

**Keywords:** obstructive sleep apnea, glucagon-like peptide-1 receptor agonists, sodium-glucose cotransporter-2 inhibitors, cardiovascular risk, obesity, type 2 diabetes

## Abstract

Obstructive sleep apnea (OSA) is a prevalent condition associated with increased cardiovascular risk, particularly in individuals with comorbid obesity and type 2 diabetes (T2D). Despite the widespread use of continuous positive airway pressure (CPAP) for OSA management, adherence remains suboptimal, and CPAP has not consistently demonstrated reductions in surrogate cardiovascular events. Recently, attention has focused on glucagon-like peptide-1 receptor agonists (GLP-1RAs) and sodium-glucose cotransporter-2 (SGLT2) inhibitors as potential therapeutic agents for mitigating cardiovascular risk in OSA patients. These agents, originally developed for T2D management, have demonstrated pleiotropic effects, including significant weight loss, blood pressure reduction, and amelioration of endothelial dysfunction and arterial stiffness, along with anti-inflammatory benefits, which may be particularly beneficial in OSA. Emerging clinical evidence suggests that GLP-1RAs and SGLT2 inhibitors can reduce OSA severity and improve daytime sleepiness, potentially reversing the adverse cardiovascular effects observed in OSA. This review explores the pathophysiological mechanisms linking OSA with cardiovascular disease and evaluates the potential therapeutic roles of GLP-1RAs and SGLT2 inhibitors in addressing cardiovascular risk in OSA patients. Further research, including long-term clinical trials, is necessary to establish the effectiveness of these therapies in reducing cardiovascular events and improving patients’ reported outcomes in this population.

## 1. Introduction

Obstructive sleep apnea (OSA) is marked by the recurrent collapse of the pharyngeal airway during sleep, leading to apneas and hypopneas [1]. This results in significant physiological disturbances, including intermittent hypoxemia, hypercapnia, and repeated sleep arousals [1]. Clinically, OSA manifests through notable symptoms such as excessive daytime sleepiness and serves as an independent risk factor for cardiovascular diseases [1,2,3]. The prevalence of OSA is substantial and increasing, affecting over 900 million people worldwide, with approximately 40% experiencing moderate to severe disease, contributing to considerable medical and economic burdens [4].

The management of OSA has traditionally centered on mechanical interventions during sleep, with CPAP therapy being the primary approach. CPAP therapy effectively lowers the apnea–hypopnea index (AHI)—a measure of apneas and hypopneas per hour of sleep—and alleviates OSA-related symptoms [5]. However, its overall efficacy is often limited by inconsistent patient adherence [6,7,8]. Despite this, randomized controlled trials have not demonstrated a reduction in primary or secondary prevention of cardiovascular events or mortality with CPAP use [9,10,11,12]. For patients who cannot tolerate or are unwilling to use CPAP, mandibular advancement devices are commonly employed, though their effectiveness is not universal [13]. Surgical interventions, such as hypoglossal nerve stimulation, may offer benefits but are invasive and typically reserved for selected cases. Currently, no pharmacological treatments for OSA have been approved.

Excess adiposity is a key modifiable risk factor contributing to both the development of OSA and its associated complications [14,15,16,17]. Substantial weight loss is widely acknowledged as beneficial in managing OSA, with clinical guidelines advocating for the treatment of obesity in affected individuals [15]. Consequently, a pharmacological intervention aimed at addressing obesity and its downstream effects—such as the mitigation of OSA symptoms, blood pressure regulation, and reduction of low-grade systemic inflammation—could offer a more comprehensive therapeutic approach, addressing aspects that mechanical treatments alone cannot fully resolve [18,19,20].

Over the past decade, glucagon-like peptide-1 receptor agonists (GLP-1RAs) have emerged as highly effective agents for reducing both blood glucose levels and body weight in individuals with type 2 diabetes (T2D), obesity, or both [21,22,23,24,25,26,27,28]. Due to their pleiotropic effects across a range of cardio-renal-metabolic comorbidities closely associated with OSA, there has been growing scientific interest in their potential role in OSA management, supported by recent randomized trials and ongoing research [24,25,29,30,31,32]. Similarly, sodium-glucose cotransporter 2 (SGLT2) inhibitors, initially developed as glucose-lowering drugs, have demonstrated significant cardiorenal benefits beyond T2D management [33,34,35,36,37,38,39,40,41,42,43]. Given their involvement in various pathophysiological pathways relevant to OSA, numerous studies have explored their potential utility in OSA management. Of note, T2D and OSA exhibit a bidirectional relationship, with OSA being highly prevalent among individuals with T2D, while also serving as a significant risk factor for the development of new-onset T2D [44,45].

Accordingly, this narrative review sought to examine the emerging insights into the pathophysiological mechanisms linking OSA to cardiovascular disease and to evaluate the potential of GLP-1 receptor agonists and SGLT2 inhibitors in attenuating cardiovascular risk among individuals with OSA.

## 2. Pathophysiological Mechanisms Underlying the Increased Cardiovascular Risk in OSA

The comprehensive methodology employed in this narrative review is detailed in the Supplementary Material (Supplementary Material Tables S1–S3).

**Figure 1:**
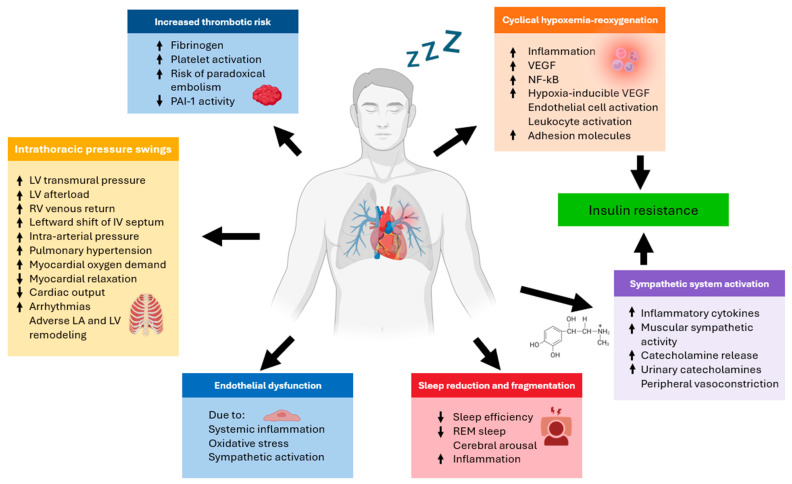
The pathogenesis of adverse cardiovascular outcomes in obstructive sleep apnea (OSA) involves several interconnected mechanisms. Sleep fragmentation and chronic intermittent hypoxia, resulting from repeated airway collapse, are key contributors. These conditions trigger increased oxidative stress and the production of reactive oxygen species, which, in turn, activate the NF-kB pathway, leading to heightened inflammation. Additionally, sympathetic nervous system activation, endothelial dysfunction, and metabolic dysregulation further exacerbate the physiological burden. Pathophysiological alterations associated with sleep apnea lead to both acute, transient electrophysiological disturbances and chronic, progressive cardiac remodeling. Each acute episode of sleep apnea induces transient peaks in arrhythmia risk due to apnea-related physiological stress. However, without a pre-existing structural cardiac substrate, these transient events alone may not reach the threshold required to initiate a sustained arrhythmia. Over time, the cumulative effects of repeated apneic episodes may contribute to the development of such substrates, increasing the likelihood of arrhythmogenic events. Collectively, these processes are believed to play a significant role in the development of cardiovascular and cerebrovascular morbidity associated with OSA. Abbreviations: LV, left ventricular; RV, right ventricular; PAI-1, plasminogen activator inhibitor type-1; REM, Rapid eye movement; VEGF, vascular endothelial growth factor. Arrows: ↓ decrease, ↑ increase.

OSA leads to acute physiological disruptions, including alterations in arterial blood gas levels, respiratory arousals, and heightened sympathetic activity, which result in transient increases in blood pressure (BP) and heart rate (HR) [46] (Figure 1). Each obstructive episode is characterized by reduced airflow, paradoxical abdominal and thoracic movements, and significant intrathoracic pressure fluctuations. The consequent decrease in ventilation causes episodic elevations in CO_2_ levels and reductions in blood oxygen saturation. The respiratory event is terminated by the spontaneous reopening of the upper airway, triggered by compensatory mechanisms or arousal from sleep, allowing oxygen saturation to return to baseline levels. However, upon reentering sleep, upper airway obstruction recurs, perpetuating this cycle throughout the night [47]. In untreated OSA, chronic exposure to intermittent hypoxemia, hypercapnia, cycles of reoxygenation, respiratory arousals, intrathoracic pressure variations, and autonomic nervous system disturbances is believed to contribute to an increased risk of cardiovascular disease (Table 1). This heightened risk is mediated through mechanisms such as BP dysregulation, endothelial dysfunction, inflammation, oxidative stress, and metabolic disruption [20].

### 2.1. Blood Pressure Disturbance

Episodic apneas and hypopneas are linked to heightened sympathetic activity at the termination of each event, leading to sharp increases in systolic and diastolic BP (BP). These surges often result in elevated nocturnal BP, which frequently persists throughout daytime [54]. The regulation of BP and HR during sleep is disrupted in direct relation to the frequency and duration of apneas and hypopneas, as well as their impact on gas exchange, sleep fragmentation, and autonomic nervous system function [55]. Intermittent hypoxemia-induced activation of the sympathetic nervous system can also contribute to dysfunction of the renin-angiotensin-aldosterone system [46]. The effects of hypoxia, however, may vary depending on its severity, with mild intermittent hypoxia potentially lowering BP, while severe hypoxia can trigger BP spikes exceeding 200 mmHg [56]. Additionally, OSA-related respiratory arousals, which reduce the time spent in deep sleep (stage N3, associated with high parasympathetic activity), are thought to play a pathogenic role, as shortened N3 sleep duration is predictive of incident hypertension [57,58]. The severity of OSA during rapid eye movement (REM) sleep, a phase characterized by diminished respiratory neuromuscular control, has been strongly correlated with the onset of hypertension and non-dipping BP patterns, where nocturnal BP falls by less than 10% compared to daytime levels [59]. This REM-specific effect is attributed to elevated sympathetic activity resulting from prolonged respiratory events and significant oxygen desaturations that typically occur in this sleep stage [60]. It has been demonstrated that successful therapeutic management of OSA usually results in significant reduction in systolic and diastolic BP, and subsequent amelioration of overall cardiovascular risk in that population [61].

### 2.2. Endothelial Dysfunction

Endothelial dysfunction, characterized by impaired endothelium-dependent vasodilation, is an early marker of atherosclerosis and an independent risk factor for cardiovascular disease and cardiovascular mortality [62]. It is considered to be a central mechanism linking OSA to adverse cardiovascular outcomes [63,64,65,66]. Non-invasive assessments of flow-mediated vasodilation have shown diminished vasodilatory responses in individuals with OSA compared to control group [67,68]. Respiratory disturbances and intermittent hypoxia associated with OSA are thought to impair endothelial function through various mechanisms, including decreased nitric oxide bioavailability and heightened oxidative stress [69,70]. Studies have demonstrated a correlation between OSA and altered blood nitrite/nitrate levels, which respond variably to CPAP therapy [71,72]. A particularly specific mechanism of endothelial dysfunction involves intermittent hypoxia, which promotes the internalization of the complement inhibitor CD59 from the endothelial surface, thereby increasing complement activation and inflammation [73].

### 2.3. Inflammation and Metabolic Dysregulation

Recurrent cycles of hypoxia and reoxygenation, which induce oxidative stress and activate inflammatory and pro-thrombotic pathways, are critical mediators of inflammation and metabolic dysfunction in atherosclerosis [74]. This phenomenon is notably observed in OSA, where intermittent hypoxia leads to the activation of the pro-inflammatory transcription factor NF-κB [74]. Studies have reported that the activation level of NF-κB correlates with the AHI and decreases following CPAP therapy, which underscores the role of intermittent hypoxia in inflammatory processes [74].

Patients with OSA exhibit elevated levels of several inflammatory and pro-thrombotic biomarkers, including intercellular adhesion molecules, tumor necrosis factor (TNF), C-reactive protein (CRP), interleukin-6 (IL-6), fibrinogen, plasminogen activator inhibitor-1 (PAI-1), activated coagulation factors, and soluble P-selectin [75]. Although some of these associations may be confounded by the pro-inflammatory effects of obesity, multiple studies have confirmed that treatment of OSA can reduce the levels of these pro-inflammatory factors [76]. Furthermore, neutrophil levels have been found to rise proportionally with AHI, with overnight HR (a proxy for cardiac autonomic balance) accounting for a significant portion of this relationship [77]. This finding supports the connection between OSA, autonomic nervous system regulation, and systemic inflammation.

Intermittent hypoxia is also implicated in metabolic dysfunction through its impact on various physiological mechanisms, including pancreatic β-cell function, adipose tissue inflammation, non-alcoholic fatty liver disease, lipolysis, and mitochondrial dysfunction, as described in prior reviews [78]. Both preclinical and clinical studies have linked intermittent hypoxia to insulin dysregulation [79]. Additionally, sleep fragmentation or deprivation, which frequently co-occurs with OSA, may exacerbate obesity, inflammation, and metabolic disturbances via multiple mechanisms including alterations in appetite-regulating hormones, impaired glucose metabolism, insulin resistance, and shifts in energy balance [80,81].

## 3. Clinical Evidence on the Association Between OSA and Cardiovascular Disease

### 3.1. OSA and Subclinical Atherosclerosis

The coexistence of coronary artery disease (CAD) and OSA is characterized by several adverse cardiovascular manifestations, including an increase in atherogenic plaque formation and heightened plaque vulnerability, reduced coronary flow reserve, episodes of acute ischemia during sleep, and an elevated incidence of myocardial infarction and sudden cardiac death. A significant marker of subclinical atherosclerosis, the coronary artery calcium (CAC) score, has been found to be notably higher in individuals with OSA. Specifically, a CAC score exceeding 400 was observed to be 40% more prevalent among those diagnosed with OSA compared to controls in a large-scale community-based study [82]. Furthermore, the progression of CAC over an 8-year period was more common in OSA patients, suggesting a link between OSA and the advancement of subclinical atherosclerosis [82].

Beyond anatomical obstructions caused by atherosclerotic plaques, the coronary artery fractional flow reserve (FFR) can fluctuate dynamically during obstructive events due to variations in intrathoracic pressure [83]. These fluctuations impact venous return, aortic pressures, and overall coronary perfusion, thereby contributing to episodes of ischemia [83]. During sleep, subclinical ischemia may arise not only from reduced oxygen delivery but also from increased oxygen demand. This increased demand is driven by elevations in diastolic and transmural pressures, which result from substantial fluctuations in intrathoracic pressure associated with obstructive breathing events [84]. Moreover, the persistent effects of hypertension, leading to cardiac hypertrophy and increased aortic stiffness further exacerbate the risk of ischemia [84].

### 3.2. Incident Cardiovascular Disease in OSA

Community-based prospective studies have demonstrated a heightened risk of CAD in individuals with OSA, a risk that remains significant even after adjusting for traditional risk factors. However, the magnitude of this association can vary based on age and sex. The Sleep Heart Health Study reported that individuals with moderate to severe OSA had a 35% greater incidence of CAD over an 8-year follow-up period compared to those without OSA [85]. Notably, the risk was even more pronounced in men under the age of 70, who exhibited a 70% higher likelihood of developing CAD if diagnosed with OSA [85]. Further evidence is provided by the Multi-Ethnic Study of Atherosclerosis, which included over 5000 participants without pre-existing cardiovascular disease at baseline [86]. Over a 7.5-year follow-up period, those with a physician-diagnosed OSA were found to have a 90% increased risk of new cardiovascular events and a 2.4-fold higher risk of mortality, underscoring the substantial impact of OSA on long-term cardiovascular health [86].

In a cohort exceeding 10,000 individuals, those experiencing clinically significant nocturnal hypoxemia exhibited an almost twofold increase in the risk of sudden cardiac death after adjustments for confounding variables. Long-term studies conducted in Spain, which monitored patients referred to sleep laboratories for periods ranging from 5 to 10 years, further support these findings [87]. Among men, severe untreated OSA was linked to a 2.9-fold greater risk of fatal cardiovascular events and a 3.2-fold higher risk of non-fatal cardiovascular events compared to controls. Similar trends have been observed in women [87]. A prospective study involving over 1000 female participants, followed for a median of 72 months, revealed that untreated moderate to severe OSA was associated with a 3.5-fold increase in cardiovascular mortality risk, even after adjusting for potential confounders [88]. These findings emphasize the significant cardiovascular risks posed by untreated OSA across different populations.

Additional research indicates that individuals with OSA experience higher rates of major acute cardiovascular events compared to those without the condition [89,90]. Of note, inadequately treated OSA has been identified as a predictor of an increased likelihood of requiring revascularization, either angioplasty or coronary artery bypass grafting [3,91]. Additionally, studies have shown that patients with OSA tend to have larger cardiac infarct sizes 3 months following myocardial infarction salvage procedures compared to those without OSA, suggesting more extensive myocardial damage and impaired recovery [92].

## 4. GLP-1 and GIP/GLP-1 Receptor Agonists and OSA

Tirzepatide is a dual receptor agonist that targets both the glucose-dependent insulinotropic polypeptide (GIP) receptor and the glucagon-like peptide-1 (GLP-1) receptor, selectively binding and activating these pathways [93]. Structurally, it is composed of an amino acid sequence that incorporates a C20 fatty diacid moiety, facilitating binding to albumin and thereby extending its half-life [93]. Clinical studies have demonstrated that treatment with tirzepatide results in substantial reductions in excess body weight, along with improvements in BP, and decreases in biomarkers associated with inflammation and vascular endothelial dysfunction [94,95]. These effects suggest that tirzepatide could potentially be an effective therapeutic option for individuals with obstructive OSA.

Recently, Malhotra and colleagues reported the findings of the very interesting SURMOUNT-OSA phase 3 clinical trials, marking a significant advancement in the integrated management of OSA and obesity [96]. These trials enrolled adults with moderate to severe OSA, defined by an apnea–hypopnea index (AHI) of 15 or more events per hour, and concurrent obesity. The study was conducted through two separate trials: the first included participants who were not undergoing CPAP therapy at the beginning of the study, while the second included those on stable CPAP treatment, incorporating a 7-day CPAP washout period at baseline and at assessments during weeks 20 and 52 [96]. A total of 469 participants were randomized to receive either tirzepatide (at doses of 10 or 15 mg) or a placebo [96].

At baseline, the mean AHI was recorded at 51.5 events per hour in Trial 1 and 49.5 events per hour in Trial 2, while the mean BMI was 39.1 and 38.7, respectively [96]. In Trial 1, after 52 weeks of treatment, the mean reduction in AHI was −25.3 events per hour (95% confidence interval [CI], −29.3 to −21.2) for participants receiving tirzepatide, compared to a reduction of −5.3 events per hour (95% CI, −9.4 to −1.1) in the placebo group, translating into in an estimated significant treatment difference of −20.0 events per hour (*p* < 0.001) [96]. In Trial 2, the mean change in AHI at week 52 was −29.3 events per hour (95% CI, −33.2 to −25.4) for the tirzepatide group, compared to −5.5 events per hour (95% CI, −9.9 to −1.2) for those receiving placebo, yielding an estimated statistically and clinically significant treatment difference of −23.8 events per hour, favoring tirzepatide (*p* < 0.001) [96].

Tirzepatide also led to significant improvements across all prespecified secondary endpoints compared to placebo [96]. That said, tirzepatide resulted in significant reductions in body weight, concentrations of high-sensitivity CRP, and sleep apnea–specific hypoxic burden—quantified through polysomnographic assessments that evaluate the frequency, duration, and severity of oxygen desaturation associated with respiratory events. Furthermore, participants experienced notable decreases in systolic BP and improved scores on the Patient-Reported Outcomes Measurement Information System (PROMIS) Short Form Sleep-related Impairment 8a (PROMIS-SRI) and the PROMIS Short Form Sleep Disturbance 8b (PROMIS-SD) scales, with higher scores reflecting greater levels of sleep impairment or disturbance. The most common adverse events associated with tirzepatide were gastrointestinal in nature and were predominantly mild to moderate in severity.

Earlier this year, O’Donnell and colleagues reported findings from a randomized proof-of-concept study that evaluated 30 obese patients newly diagnosed with moderate to severe OSA [97]. The study excluded individuals with T2D, heart failure, or unstable cardiovascular disease. Participants were assigned to one of 3 treatment groups for a 24-week period: CPAP therapy (Group A), liraglutide monotherapy (Group B), or a combination of CPAP and liraglutide (Group C) [97]. The results showed that CPAP, whether administered alone or in combination with liraglutide, led to a significantly greater reduction in the AHI compared to liraglutide monotherapy [97]. Specifically, the mean decrease in AHI was 45 and 43 events per hour for CPAP and combination therapy, respectively, versus 12 events per hour for liraglutide alone (*p* < 0.05) [97]. While liraglutide and combination therapy both induced notable weight loss, only CPAP monotherapy significantly reduced vascular inflammation, as evidenced by a decrease in the aortic wall target-to-background ratio (*p* = 0.010) [97]. This improvement was also associated with enhanced endothelial function and lower levels of CRP. Furthermore, both CPAP and combination therapy were effective in reducing low-attenuation coronary artery plaque volume—a marker of unstable atherosclerotic plaque [97]. However, no significant changes in this parameter were observed with liraglutide monotherapy, highlighting the distinct vascular benefits of CPAP in this patient cohort [97].

Jiang and colleagues conducted a two-center, prospective randomized controlled trial involving 90 patients with T2D and severe OSA [98]. Participants were assigned to receive either a combination of CPAP and drug therapy that included liraglutide or CPAP and drug therapy without liraglutide [98]. The study found that adding liraglutide resulted in significant reductions in BMI, AHI, and mean systolic BP compared to the control group (*p* < 0.05) [98]. Additionally, patients in the liraglutide group showed a significantly higher minimum oxygen saturation level after 3 months of treatment (*p* < 0.05), indicating improved nocturnal oxygenation [98]. Importantly, the incidence of adverse effects did not differ significantly between the two groups (*p* > 0.05), suggesting liraglutide was well-tolerated within the study population [98].

Blackman et al. presented results from a randomized, double-blind trial involving participants with obesity, but without T2D, who had either moderate (AHI 15–29.9 events/h) or severe (AHI ≥ 30 events/h) OSA and were either unwilling or unable to use CPAP [99]. The participants were randomized to receive either liraglutide 3.0 mg (n = 180) or placebo (n = 179) over a 32-week period [99]. Liraglutide significantly outperformed placebo in reducing AHI, with an estimated treatment difference of −6.1 events per hour (*p* = 0.015) [99]. Additionally, liraglutide led to a substantially greater reduction in body weight, showing an estimated treatment difference of −4.2% compared to placebo (*p* < 0.0001) [99]. Post hoc analyses further identified a significant association between the degree of weight loss and improvements in OSA severity (*p* < 0.01 for all measures) [99]. Moreover, liraglutide treatment produced more marked reductions in both HbA1c and systolic BP (SBP) relative to placebo [99]. The safety profile of liraglutide at a 3.0 mg dose was consistent with observations from lower doses, such as 1.8 mg, indicating tolerability across different dosages [99].

Gomez-Peralta et al. conducted a single-center retrospective study involving 58 obese adults with T2D who had been receiving liraglutide treatment for at least 3 months prior to study enrollment [100]. The results revealed significant reductions in Epworth Sleepiness Scale (ESS) scores at both 1 month (−1.3 ± 2.8, *p* < 0.001) and 3 months (−1.5 ± 3.0, *p* < 0.001) after initiating liraglutide therapy, indicating improvements in daytime sleepiness [100]. Furthermore, after 3 months of treatment, participants demonstrated substantial improvements in various anthropometric and metabolic parameters [100]. There were significant decreases in body weight (*p* < 0.001), BMI (*p* < 0.001), and circumferential measurements of the waist (*p* < 0.001) and neck (*p* < 0.005) [100]. Additionally, liraglutide therapy was associated with marked reductions in HbA1c levels (*p* < 0.001), mean blood glucose (*p* < 0.001), fasting plasma glucose (*p* < 0.001), triglycerides (*p* < 0.01), and total cholesterol (*p* < 0.001), underscoring its efficacy in improving glycemic control and lipid profiles [100].

Beyond the improvements in objective measures observed with GLP-1RAs in individuals with OSA, a randomized trial has also shown that exenatide is associated with a significant reduction in objective sleepiness among obese patients with T2D, independent of their HbA1c levels, within 4 weeks of treatment [101]. Additionally, a single-center, open-label, prospective phase 4 randomized controlled trial is currently underway, involving 132 patients newly diagnosed with OSA (AHI ≥ 15 events/h), who also have obesity and T2D. Participants will be randomly allocated into one of four treatment groups in equal proportions for a 26-week period: (i) liraglutide at a daily dose of 1.8 mg, (ii) a combination of liraglutide 1.8 mg daily and CPAP, (iii) CPAP monotherapy as the standard care, or (iv) a control group receiving no intervention [102]. This trial aims to elucidate the potential dose–response relationship of liraglutide in managing OSA, particularly in the context of coexisting obesity and T2D [102].

A real-world study investigating adherence to GLP-1RAs found that the majority of patients with OSA, irrespective of the presence of T2D, failed to remain adherent to therapy for a full year, with adherence rates of 48.8% among those with T2D and 32.4% among those without T2D [103]. Notably, OSA patients with comorbid T2D exhibited higher adherence and were less likely to discontinue GLP-1RA treatment than those without T2D [103]. It is important to highlight, however, that overall adherence rates to GLP-1RAs in patients with OSA are comparable to those observed in the general population prescribed these medications [104]. Reasons for low adherence rates may be severe gastrointestinal adverse events, parenteral route of administration, high cost of treatment, or lack of adequate drug supplies for maintenance of treatment.

**Figure 2:**
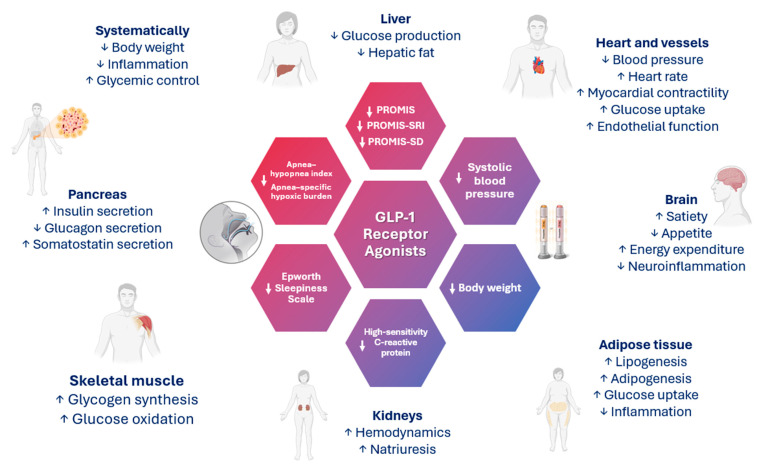
The pleiotropic effects of glucagon-like peptide-1 receptor agonists (GLP-1RAs) and their potential role in management of obstructive sleep apnea (OSA). Evidence from randomized trials indicates that GLP-1RAs significantly reduce body weight in individuals with type 2 diabetes (T2D), overweight or obesity, and/or prediabetes. Notably, in individuals with obstructive sleep apnea (OSA), weight loss emerged as the primary factor linked to improvements in the apnea–hypopnea index (AHI) and other sleep-related measures, underscoring the pivotal role of body weight reduction in ameliorating OSA severity and associated sleep disturbances. The widely acknowledged pleiotropic effects of GLP-1RAs—including the normalization of neurogenesis and synaptic plasticity, protection against neuronal apoptosis, and reduction of oxidative stress—suggest their potential influence on various neurological functions. Given the presence of GLP-1 receptors in hypothalamic nuclei involved in regulating sleep and wakefulness, GLP-1RAs may exert direct effects on sleep-related outcomes. These effects may extend beyond sleepiness to encompass cognitive functions such as memory, as well as emotional parameters like anxiety and depression. Abbreviations: PROMIS, Patient-Reported Outcomes Measurement Information System, PROMIS-SRI, Short. Form Sleep-related Impairment 8a; PROMIS-SD, PROMIS Short Form Sleep Disturbance 8b. Arrows: ↓ decrease, ↑ increase.

Figure 2 depicts the diverse, pleiotropic effects of GLP-1 receptor agonists and their potential therapeutic role in the management of OSA. Table 2 summarizes the main characteristics and outcomes of the available clinical studies.

## 5. SGLT2 Inhibitors and OSA

**Figure 3:**
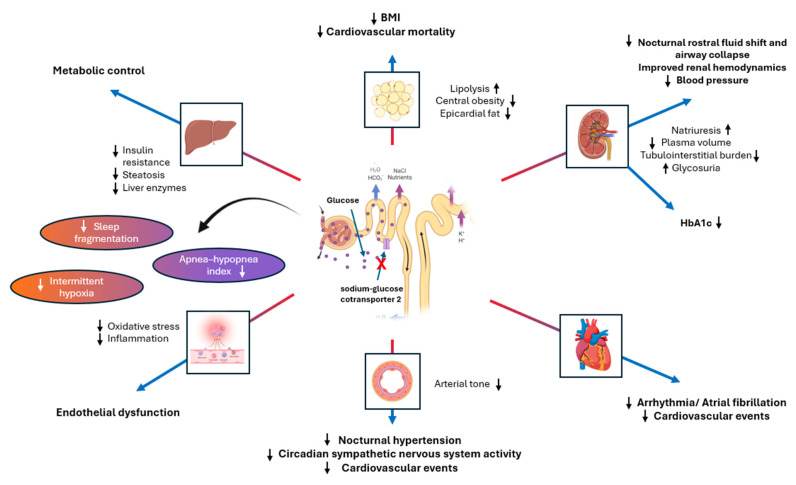
The potential therapeutic mechanisms of SGLT2 inhibitors in obstructive sleep apnea (OSA) are multifaceted. SGLT2 inhibition may reduce the apnea–hypopnea index (AHI) and cardiovascular mortality by enhancing lipolysis and reducing central obesity and epicardial fat. It may also mitigate nocturnal rostral fluid shift and protect the airway by promoting natriuresis, thereby lowering plasma volume and tubulointerstitial burden. Additionally, SGLT2 inhibitors could reduce the incidence of arrhythmias and cardiovascular risk by decreasing oxygen demand and fibrosis. They may also mitigate nocturnal hypertension, attenuate circadian sympathetic nerve activity, and reduce stroke risk by preventing activation of sympathetic nervous system and decreasing arterial tone. Furthermore, SGLT2 inhibitors may improve endothelial function by reducing oxidative stress and inflammation, while addressing metabolic dysregulation by decreasing insulin resistance, steatosis, and liver enzyme levels. Abbreviations: BMI, body-mass index. ×: inhibition; Arrows: ↓ decrease, ↑ increase.

SGLT2 inhibitors represent another class of glucose-lowering agents with promising potential for reducing cardiovascular risk in the context of OSA (Figure 3).

In a post hoc analysis of the hallmark VERTIS CV trial by Wojeck et al., patients ≥ 40 years with T2D and atherosclerotic cardiovascular disease were randomized to receive either ertugliflozin (5 or 15 mg) or placebo [105] (Table 3). Of the 8246 patients enrolled, 7697 (93.3%) did not have OSA at baseline. The incidence rate of OSA was 1.44 per 1000 person-years in the ertugliflozin group compared to 2.61 per 1000 person-years in the placebo group, corresponding to a 48% relative risk reduction [105]. These results suggest that ertugliflozin nearly halved the incidence of OSA in patients with T2D and cardiovascular disease [105].

In an open-label trial, 16 patients with T2D and OSA were randomized to receive empagliflozin (10 mg), dapagliflozin (5 mg), or luseogliflozin (2.5 mg) in conjunction with CPAP therapy [106]. After 3 months of CPAP therapy, a marked reduction in the AHI was observed in both the SGLT2 inhibitor and non-SGLT2 inhibitor groups when compared to baseline [106]. Despite this improvement in AHI, there were no significant changes in serum HbA1c levels in either group post-therapy [106]. Notably, in the SGLT2 inhibitor group, body weight, and BMI increased significantly after 3 months of CPAP therapy, an effect that was not observed in the non-SGLT2 inhibitor group [106].

In the largest analysis conducted to date, Neeland et al. investigated the effects of empagliflozin on the incidence of OSA, as well as its impact on metabolic, cardiovascular, and renal outcomes in participants with and without OSA, within the context of the EMPA-REG OUTCOME trial [107]. A total of 391 patients with T2D and cardiovascular disease, who also had OSA, were randomized to receive empagliflozin (10 or 25 mg) or placebo daily in addition to standard care [107]. Over a median follow-up of 3.1 years, empagliflozin led to comparable placebo-adjusted reductions in HbA1c, waist circumference, and systolic BP, irrespective of OSA status [107]. However, weight reduction was more pronounced in individuals with OSA compared to those without. In the placebo group, patients with baseline OSA exhibited a 1.2- to 2.0-fold higher incidence of 3-point major adverse cardiovascular events, cardiovascular death, heart failure hospitalization, and worsening nephropathy compared to those without OSA [107]. Empagliflozin significantly lowered the risk of these adverse outcomes, regardless of OSA status (P-interaction > 0.05 for all outcomes) [107]. Moreover, 50 patients were newly diagnosed with OSA within 7 days of discontinuing treatment, with a lower incidence observed in the empagliflozin group (hazard ratio 0.48) [107].

In a randomized controlled trial involving 36 patients newly diagnosed with T2D and OSA, the effects of dapagliflozin combined with metformin were compared to glimepiride combined with metformin over 24 weeks [108]. The study evaluated changes in fasting plasma glucose (FPG), postprandial blood glucose (PPG), HbA1c, fasting insulin levels, homeostasis model assessment of insulin resistance (HOMA-IR), lipid profile, BMI, BP, AHI, minimum oxygen saturation (LSpO2), and ESS score [108]. In the dapagliflozin group, significant reductions in triglycerides (TG), and in SBP and DBP were observed, alongside a notable increase in high-density lipoprotein cholesterol (HDL-C) (*p* < 0.05). This group also demonstrated a significant decrease in AHI, improvement in LSpO2, and a reduction in ESS score (*p* < 0.05), outcomes not observed in the control group [108]. Both groups experienced significant improvements in blood glucose, HbA1c, HOMA-IR, and BMI, though these effects were more pronounced in the dapagliflozin group [108].

Sawada et al. reported findings of a smaller retrospective cohort study with 18 patients with T2D and OSA [109]. A significant reduction in the AHI was observed with SGLT2 inhibitors, decreasing from 31.9 ± 18.0 to 18.8 ± 11.5 events per hour (*p* = 0.003) [109]. Significant decreases in HbA1c, body weight, and BMI were also noted, while BP remained unaffected [109]. A significant relationship between the reduction in AHI and the pre-treatment AHI was identified through Pearson correlation analysis [109]. A recently published cohort study involving 514 consecutive elderly outpatients with heart failure, T2D, and OSA, who were not receiving CPAP therapy, reported significant improvements in polygraphic parameters in the group treated with SGLT2 inhibitors [111]. From baseline to follow-up, this group exhibited marked reductions in AHI (*p* < 0.0001), oxygen desaturation index (ODI) (*p* < 0.0001), and total time with oxygen saturation below 90% (TC90) (*p* < 0.0001), alongside a significant improvement in mean SpO2 (91.3% vs. 93.8%; *p* < 0.0001) [111]. These positive changes were not observed in the untreated cohort. The use of SGLT2 inhibitors in patients with heart failure and mixed-type sleep apnea not undergoing CPAP therapy was found to significantly improve polygraphic outcomes [111]. A similar beneficial effect on ODI has also been demonstrated by Furukawa et al. [110].

Finally, a patient-level pooled analysis of the DAPA-HF and DELIVER trials, which randomized heart failure patients across the full spectrum of left ventricular ejection fraction to dapagliflozin 10 mg or placebo, demonstrated that the primary outcome—worsening heart failure or cardiovascular death—occurred more frequently in participants with sleep apnea compared to those without (16.0 per 100 person-years in participants with sleep apnoea, compared to 10.6 per 100 person-years in those without) [112]. However, dapagliflozin was found to reduce the risk of this primary endpoint similarly in both groups, with no significant difference in treatment effect between those with sleep apnea and those without [112]. Taken together, these data indicate that the absence of a significant interaction effect suggests that dapagliflozin’s benefits on heart failure outcomes are consistent, thereby supporting its use in this patient population.

## 6. Conclusions and Future Directions

Despite the encouraging data currently available, the existing evidence remains preliminary due to the limited number of randomized controlled trials (RCTs) and the relatively small sample sizes of the included participants. With the ongoing emergence of promising evidence regarding the efficacy of SGLT2 inhibitors and GLP-1RAs in addressing a range of cardiometabolic disorders, the field of sleep medicine may be on the brink of a significant transformation in the management of OSA—especially in individuals with coexisting overweight or obesity. Of course, we should mention that OSA is not currently included in the cardio-kidney-metabolic (CKM) syndrome definition proposed by American Heart Association in November 2023. However, future amendments to that statement should also include OSA, as it should be considered the “sleep” complication of dysmetabolism, closely related to adverse cardiovascular events through both shared and distinct mechanisms [113]. While the standard treatment with CPAP has not consistently shown meaningful improvements in long-term health outcomes, weight loss interventions, such as those demonstrated in the SURMOUNT-OSA trial, have produced notable reductions in both OSA severity and daytime sleepiness. These outcomes could rival those achieved with CPAP therapy alone, while also yielding additional benefits in critical cardiometabolic risk factors. Although these findings suggest that weight loss through incretin-based therapies may serve as a viable first-line treatment for OSA in individuals with overweight or obesity, further investigation is required. Specifically, direct head-to-head comparative effectiveness trials focusing on key metrics such as reductions in AHI, sleepiness, sleep-related quality of life, and BP are necessary to substantiate this approach. In addition, it appears reasonable that individuals with OSA and concomitant heart failure, across the wide range of left ventricular ejection fraction, may benefit from treatment with SGLT2 inhibitors, due to their multiple, pleiotropic benefits, regardless of baseline glycemic control or concurrent T2D status. Moreover, longer-term studies are eagerly anticipated to assess patients’ adherence over time and to determine whether such interventions can lead to superior reductions in hard cardiovascular endpoints, thereby providing definitive guidance for managing this growing and challenging patient population.

## Figures and Tables

**Table 1:** Studies investigating sleep apnea-associated hypoxic burden and adverse cardio-renal and vascular outcomes.

Study (Year)	Cohort	Number of Patients	Age (Years); Men (%)	Hypoxic Burden (%min/h)	Follow-Up Duration	Outcome; Number of Events	Findings
Azarbarzin, 2019 [48]	MrOS	2743	76.3 ± 5.5; 100	58.4 ± 52.9	10 ± 3.5 years	Cardiovascular mortality; 440	High hypoxic burden was linked to increased cardiovascular mortality
	SHHS	5111	63.7 ± 10.9; 47.2	50.2 ± 57.3	10.9 ± 3.1 years	Cardiovascular mortality; 313	
Blanchard, 2021 [49]	Pays de la Loire Sleep Cohort	3597	58 (48–67); 63	Not reported	5.9 (3.5–8.4) years	First incident stroke; 83 (70 ischemic, including TIA)	Increased hypoxic burden was associated with stroke
Azarbarzin, 2020 [50]	SHHS	4881	63.6 ± 11.1; 45.6	62 ± 64.7 in men; 37 ± 39.4 in women	10.4 ± 3.4 years	Incident heart failure; 543	Increased hypoxic burden was associated with incident heart failure in men
	MrOS	2653	76.2 ± 5.4; 100	57.3 ± 53	8.8 ± 2.8 years	Incident heart failure; 145	
Kim, 2020 [51]	MESA	2055	68.4 ± 9.1; 46	56.6 ± 65.9	Cross-sectional	Systolic and diastolic blood pressure	Increased hypoxic burden was associated with higher diastolic blood pressure
Jackson, 2021 [52]	MESA	1895	68.2 ± 9.1; 46	56.5 ± 65.1	Cross-sectional	Prevalent moderate-to-severe chronic kidney disease	Increased hypoxic burden was associated with higher prevalence of moderate-to-severe chronic kidney disease
Trzepizur, 2022 [53]	Pays de la Loire Sleep Cohort	5358	60 (51–69); 63.7	32 (13–71)	78 (52–109) months	MACE; 592	Increased hypoxic burden was associated with an increased risk of MACE

Values are presented as mean ± standard deviation or median (interquartile range). Abbreviations used MACE, major adverse cardiovascular events; MESA Multi-Ethnic Study of Atherosclerosis; MrOS, Outcomes of Sleep Disorders in Older Men study; SHHS, Sleep Heart Health Study; TIA, transient ischemic attack.

**Table 2:** Studies evaluating the effect of glucagon-like peptide-1 receptor agonists on obstructive sleep apnea.

Author/Study	Study Design	Participants	Intervention	Primary Endpoint	Outcomes
Malhotra, 2024 [96]	SURMOUNT-OSA; Phase 3, double-blind, randomized, controlled trials	In Trial 1, 234 participants who were not undergoing CPAP treatment at baseline were enrolled, while Trial 2 included 235 participants who were already receiving CPAP therapy at the start of the study. Patients with baseline diabetes were excluded	Maximum tolerated dose of tirzepatide (10 mg or 15 mg) or placebo for 52 weeks	The change in the AHI (the number of apneas and hypopneas during an hour of sleep) from baseline	By week 52 in Trial 1, AHI decreased by an average of 25.3 events per hour (95% CI, −29.3 to −21.2) in the tirzepatide group compared to a reduction of 5.3 events per hour (95% CI, −9.4 to −1.1) with placebo, resulting in a treatment difference of −20.0 events per hour (95% CI, −25.8 to −14.2). In Trial 2, the AHI reduction with tirzepatide was 29.3 events per hour (95% CI, −33.2 to −25.4), while placebo showed a reduction of 5.5 events per hour (95% CI, −9.9 to −1.2), yielding a treatment difference of −23.8 events per hour (95% CI, −29.6 to −17.9). Tirzepatide significantly improved all prespecified secondary outcomes compared to placebo
O’Donnell, 2024 [97]	Randomized proof-of-concept study	30 obese patients with newly diagnosed moderate to severe OSA. Those with T2D, heart failure, or unstable cardiovascular disease were excluded	CPAP (group A), liraglutide (group B) and combination therapy (group C) for 24 weeks	The change in the AHI and several cardiometabolic parameters	CPAP therapy, both alone and in combination, produced a significantly greater reduction in the AHI compared to liraglutide alone, with mean decreases of 45 and 43 events per hour, respectively, versus 12 events per hour (*p* < 0.05). While both liraglutide and combination therapy resulted in substantial weight loss, only CPAP monotherapy led to a significant reduction in vascular inflammation, as indicated by a decrease in the aortic wall target-to-background ratio (*p* = 0.010). This was accompanied by improvements in endothelial function and reductions in C-reactive protein levels. Additionally, low-attenuation coronary artery plaque volume, a marker of unstable plaque, decreased with both CPAP therapy and combination treatment, but no significant changes were observed with liraglutide monotherapy.
Jiang, 2023 [98]	Two-center, prospective randomized controlled trial	90 patients with T2D and severe OSA	CPAP and drug treatment including liraglutide or CPAP and drug treatment without liraglutide)	Demographic and clinical characteristics, along with indices of sleep-disordered breathing and cardiac function, as well as adverse effects, were assessed and compared between the two groups both at baseline and after a 3-month follow-up	Liraglutide was associated with significant reductions in BMI, AHI, and mean systolic blood pressure compared to the control group (*p* < 0.05). Additionally, the liraglutide group exhibited a significantly higher minimum oxygen saturation after 3 months of follow-up (*p* < 0.05). There were no significant differences between the groups regarding the incidence of side effects (*p* > 0.05)
Sprung, 2020 [102]	Single-centered, open-labelled, prospective, phase 4 randomized controlled trial	132 patients with newly diagnosed OSA (AHI ≥ 15 events/hour), and existing obesity and T2D	Participants will be randomly assigned in equal proportions to one of four treatment groups for a duration of 26 weeks: (i) liraglutide at a daily dose of 1.8 mg, (ii) liraglutide 1.8 mg daily combined with CPAP, (iii) CPAP alone as standard care, or (iv) a control group receiving no treatment	The change in OSA severity, determined by AHI	Ongoing
Blackman, 2016 [99]	Randomized, double-blind trial	Participants with obesity, without T2D who had moderate (AHI 15–29.9 events/h) or severe (AHI ≥ 30 events/h) OSA and were unwilling/unable to use CPAP	Liraglutide 3.0 mg (n = 180) or placebo (n = 179) for 32 weeks	The change in the AHI from baseline to week 32, assessed using the 2007 criteria recommended by the American Academy of Sleep Medicine. According to this definition, hypopnea events were scored based on a reduction of at least 30% in nasal pressure signal excursions from baseline, accompanied by a desaturation of at least 4% from the pre-event baseline	After 32 weeks, the reduction in the AHI was significantly greater with liraglutide compared to placebo,, resulting in an estimated treatment difference of −6.1 events per hour (*p* = 0.015). Liraglutide also led to a greater percentage of weight loss compared to placebo, with an estimated treatment difference of −4.2% (*p* < 0.0001). Post hoc analyses revealed a significant correlation between the extent of weight loss and improvements in OSA outcomes (*p* < 0.01 for all). Furthermore, liraglutide resulted in more pronounced reductions in both HbA1c and SBP compared to placebo. The safety profile of liraglutide at 3.0 mg was consistent with that observed at doses of 1.8 mg or lower.
Gomez-Peralta, 2015 [100]	Single-center retrospective study	58 obese adult subjects with T2D	Liraglutide treatment at least 3 months before study inclusion	Epworth Sleepiness Scale (ESS), anthropometric parameters, glucose-control and several metabolic parameters	Significant reductions in the ESS scores were observed at both 1 month (−1.3 ± 2.8, *p* < 0.001) and 3 months (−1.5 ± 3.0, *p* < 0.001) following the initiation of liraglutide treatment. Additionally, after 3 months of liraglutide therapy, there were notable improvements in body weight (*p* < 0.001), BMI (*p* < 0.001), waist circumference (*p* < 0.001), and neck circumference (*p* < 0.005), as well as significant reductions in HbA1c (*p* < 0.001), mean blood glucose levels (*p* < 0.001), fasting plasma glucose (*p* < 0.001), triglycerides (*p* < 0.01), and total cholesterol (*p* < 0.001).
Idris, 2013 [101]	Placebo-controlled single-blind study	80 obese patients with T2D excessive daytime sleepiness	Exenatide for 22 weeks (5 μg twice-daily dose by subcutaneous injection and increased to 10 μg twice daily within 4 weeks of treatment initiation)	Wakefulness and sleep latency test, Epworth score, driving performance, depression score, fasting glucose and HbA1c	Exenatide is linked to a substantial decrease in objective sleepiness among obese patients with T2D, regardless of their HbA1c levels.

Abbreviations: AHI, apnea–hypopnea index; BMI, body mass index; CPAP, continuous positive airway pressure; HbA1c, glycated hemoglobin; ESS, Epworth sleepiness scale; GLP-1RA, glucagon-like peptide-1 receptor agonists; OSA, obstructive sleep apnea syndrome; SBP, systolic blood pressure; T1D, type 1 diabetes; T2D, type 2 diabetes.

**Table 3:** Studies evaluating the effect of sodium-glucose cotransporter-2 inhibitors on obstructive sleep apnea.

Author/Study	Study Design	Participants	Intervention/Exposure	Primary Endpoint	Outcomes
Wojeck, 2022 [105]	Randomized controlled trial	Patients ≥ 40 years with T2D and atherosclerotic cardiovascular disease	Ertugliflozin (5 or 15 mg) or placebo	The composite of major adverse cardiovascular events	The incidence rate of OSA was 1.44 per 1000 person-years in the ertugliflozin group, compared to 2.61 per 1000 person-years in the placebo group, representing a 48% relative reduction in risk. In the VERTIS CV trial, ertugliflozin reduced the incidence of OSA by nearly 50% in patients with T2D and cardiovascular disease
Kusunoki, 2021 [106]	Open label trial	16 patients with T2D and OSA	Empagliflozin (10 mg), dapagliflozin (5 mg) and luseogliflozin (2.5 mg) along with CPAP therapy	Change in body weight, BMI, serum HbA1c level, lipid profile, liver function parameters, serum uric acid, and AHI	After 3 months of CPAP therapy, a significant reduction in the AHI was observed in both the SGLT2 inhibitors and non-SGLT2 inhibitors groups compared to baseline. In the SGLT2 inhibitor group, both body weight and BMI increased significantly after 3 months of CPAP therapy, an effect not seen in the non-SGLT2 inhibitor group
Neeland, 2020 [107]	Double-blind, placebo-controlled randomized trial (EMPA-REG OUTCOME)	391 OSA patients with T2D and cardiovascular disease	Empagliflozin (10 and 25 mg) or placebo daily in addition to standard of care	The composite outcome 3P-MACE (death as a result of CV causes, nonfatal myocardial infarction, or nonfatal stroke), with secondary outcomes including hospitalization for heart failure, all-cause mortality, and incident or worsening nephropathy	Over a median follow-up of 3.1 years, empagliflozin produced comparable placebo-adjusted reductions in HbA1c, waist circumference, and systolic blood pressure, irrespective of OSA status. However, it had a greater impact on weight reduction, for individuals with OSA versus those without OSA. Empagliflozin significantly reduced the risk of these adverse outcomes regardless of OSA status (*p*-interaction > 0.05 for all outcomes). 50 patients were newly diagnosed with OSA within 7 days of discontinuing medication, with a lower incidence reported in the empagliflozin group (hazard ratio 0.48)
Tang, 2019 [108]	Randomized controlled trial	36 patients with newly-diagnosed T2D and OSA	Dapagliflozin and metformin versus glimepiride and metformin for 24 weeks	Changes in fasting plasma glucose (FPG), postprandial blood glucose (PPG), (HbA1c), fasting insulin levels, homeostasis model assessment of insulin resistance (HOMA-IR), lipid profile, BMI, blood pressure, AHI, minimum oxygen saturation (LSpO2), ESS score	In the dapagliflozin group, significant reductions were observed in triglycerides (TG), SBP, and DBP, while high-density lipoprotein cholesterol (HDL-C) showed a significant increase (*p* < 0.05). This group exhibited a marked reduction in the AHI, an improvement in minimum oxygen saturation (LSpO2), and a decrease in the ESS score (*p* < 0.05), changes not mirrored in the control group. Both groups experienced significant reductions in blood glucose, HbA1c, HOMA-IR, and BMI, though these improvements were more pronounced in the dapagliflozin group
Sawada, 2018 [109]	Retrospective cohort study	18 patients with T2D and OSA	SGLT2 inhibitors	HbA1c, body weight, BMI, blood pressure and AHI were evaluated before and after SGLT2 inhibitors administration	A significant reduction in the AHI was observed with SGLT2 inhibitors, decreasing from 31.9 ± 18.0 to 18.8 ± 11.5 events per hour (*p* = 0.003). Significant decreases in HbA1c, body weight, and BMI were also noted, while blood pressure remained unaffected. A significant relationship between the reduction in AHI and the pre-treatment AHI was identified through Pearson correlation analysis
Furukawa, 2018 [110]	Open-label, single-arm, multicentre trial	30 patients with T2D and sleep-disordered breathing	Dapagliflozin (5 mg) once daily for 24 weeks	Change in at least five 3% oxygen desaturation index (ODI) events per hour	An improvement in the 3% ODI was evident in patients with moderate to severe sleep-disordered breathing (*p* = 0.017). However, this improvement was not observed in individuals with mild sleep-disordered breathing
Armentaro, 2024 [111]	Observational cohort study	514 consecutive elderly outpatients with heart failure, T2D and OSA not on CPAP therapy	SGLT2 inhibitors	Change in AHI	The SGLT2 inhibitors group demonstrated significant improvements in polygraphic parameters from baseline to follow-up, with reductions in the AHI (*p* < 0.0001), oxygen desaturation index (ODI) (*p* < 0.0001), and total time with oxygen saturation below 90% (TC90) (*p* < 0.0001). Mean SpO2 also improved (91.3 vs. 93.8; *p* < 0.0001). These benefits were not observed in the untreated population. SGLT2 inhibitors in patients with heart failure and mixed-type sleep apnoea not receiving CPAP therapy significantly enhanced polygraphic parameters
Butt, 2024 [112]	Patient-level pooled analysis of DAPA-HF and DELIVER trials	11,005 patients with in HFrEF and HFmrEF/HFpEF	Dapagliflozin (10 mg)	A composite of worsening heart failure or cardiovascular death	The primary outcome occurred at a rate of 16.0 per 100 person-years in participants with sleep apnoea, compared to 10.6 per 100 person-years in those without (adjusted HR 1.29 [95% CI, 1.10–1.52]). Dapagliflozin reduced the risk of the primary endpoint similarly in both patients with sleep apnea (HR 0.78 [95% CI, 0.59–1.03]) and those without (HR 0.79 [95% CI, 0.72–0.87]), with no significant interaction effect (*p*-interaction = 0.93)

Abbreviations: AHI, apnea–hypopnea index; BMI, body mass index; CPAP, continuous positive airway pressure; DBP, diastolic blood pressure; HbA1c, glycated hemoglobin; HFrEF, heart failure with reduced ejection fraction; HFmrEF, heart failure with mildly reduced ejection fraction; HFpEF, heart failure with preserved ejection fraction; HR, hazard ratio; ESS, Epworth sleepiness scale; OSA, obstructive sleep apnea syndrome; SBP, systolic blood pressure; SGLT2 inhibitors, sodium-glucose cotransporter 2 inhibitors; T2D, type 2 diabetes.

## Data Availability

No new data were created or analyzed in this study.

## Supplementary Materials

The following supporting information can be downloaded at: https://www.mdpi.com/article/10.3390/biomedicines12112503/s1, Table S1: Search strategy PubMed; Table S2: Search strategy Scopus; Table S3: Search strategy Cochrane Library.
