# Supplementary material for: Mitigating Increased Cardiovascular Risk in Patients with Obstructive Sleep Apnea Using GLP-1 Receptor Agonists and SGLT2 Inhibitors: Hype or Hope?
Source: Biomedicines. 2024 Nov 1;12(11):2503. doi: 10.3390/biomedicines12112503 (PMC11591904; doi:10.3390/biomedicines12112503)
Supplement: Supplementary file 1 [file biomedicines-12-02503-s001.zip › biomedicines-3286855-supplementary.pdf]

Supplementary material

Mitigating increased cardiovascular risk in patients with obstructive sleep apnea using GLP-1 receptor agonists and SGLT2 inhibitors: hype or hope?

Methodology

A comprehensive and independent literature search was conducted in duplicate across MEDLINE (via PubMed), Scopus, and Cochrane Library up to October 15, 2024 **(Supplementary Tables S1-S3).** The search strategy was unrestricted by language, encompassing both free-text terms and Medical Subject Headings (MeSH). The core search terms included "obstructive sleep apnea," "GLP-1 receptor agonists," and "SGLT-2 inhibitors". To ensure thoroughness, additional manual searches were performed using Epistemonikos and Google Scholar to identify any relevant studies recently published but not yet indexed in MEDLINE. Furthermore, reference lists of retrieved articles were examined, and citation tracking was utilized as part of a snowball sampling approach to identify additional pertinent studies.

Exclusions were applied to preprint articles, as well as conference speeches, oral presentations, posters, case reports, editorials, and commentaries. Duplicate records were identified and removed, followed by an initial screening of the titles, abstracts, and keywords of the remaining studies to determine their relevance to the review's objectives. Full-text assessments were conducted for studies deemed potentially relevant, accompanied by a critical evaluation. Discrepancies during this process were resolved through discussion or, if necessary, by consulting a third, more experienced author for adjudication.

**Supplementary Table S1. Search strategy PubMed**
("obstructive sleep apnoea"[All Fields] OR "sleep apnea, obstructive"[MeSH Terms] OR ("sleep"[All Fields] AND "apnea"[All Fields] AND "obstructive"[All Fields]) OR "obstructive sleep apnea"[All Fields] OR ("obstructive"[All Fields] AND "sleep"[All Fields] AND "apnea"[All Fields])) AND ("glp 1ra*"[All Fields] OR "glucagon like peptide 1 receptor"[MeSH Terms] OR (("glucagon like peptide 1 receptor"[MeSH Terms] OR ("glucagon like"[All Fields] AND "peptide 1"[All Fields] AND "receptor"[All Fields]) OR "glucagon like peptide 1 receptor"[All Fields] OR "glucagon like peptide 1 receptor"[All Fields]) AND "agonist*"[All Fields]) OR ("exenatide"[MeSH Terms] OR "exenatide"[All Fields] OR "exenatide s"[All Fields]) OR ("lixisenatide"[Supplementary Concept] OR "lixisenatide"[All Fields]) OR ("liraglutid"[All Fields] OR "liraglutide"[MeSH Terms] OR "liraglutide"[All Fields] OR "liraglutide s"[All Fields]) OR ("dulaglutide"[Supplementary Concept] OR "dulaglutide"[All Fields]) OR ("semaglutide"[Supplementary Concept] OR "semaglutide"[All Fields]) OR ("rglp 1 protein"[Supplementary Concept] OR "rglp 1 protein"[All Fields] OR "albiglutide"[All Fields]) OR ("efpeglenatide"[Supplementary Concept] OR "efpeglenatide"[All Fields]) OR ("tirzepatide"[Supplementary Concept] OR "tirzepatide"[All Fields]) OR (("sodium-glucose"[All Fields] AND "co-transporter-2"[All Fields] AND "inhibitor*"[All Fields]) OR "sodium glucose transporter 2 inhibitors"[MeSH Terms] OR ("SGLT-2"[All Fields] AND "inhibitor*"[All Fields]) OR (("sodium glucose transport proteins"[MeSH Terms] OR ("sodium-glucose"[All Fields] AND "transport"[All Fields] AND "proteins"[All Fields]) OR "sodium glucose transport proteins"[All Fields] OR ("sodium"[All Fields] AND "glucose"[All Fields] AND "cotransporter"[All Fields]) OR

"sodium glucose cotransporter"[All Fields]) AND "2"[All Fields]) OR ("SGLT2"[All Fields] AND "inhibitor*"[All Fields]) OR (("sodium glucose transport proteins"[MeSH Terms] OR ("sodium-glucose"[All Fields] AND "transport"[All Fields] AND "proteins"[All Fields]) OR "sodium glucose transport proteins"[All Fields] OR ("sodium"[All Fields] AND "glucose"[All Fields] AND "cotransporter"[All Fields]) OR "sodium glucose cotransporter"[All Fields]) AND "2"[All Fields] AND "inhibitor*"[All Fields]) OR ("empagliflozin"[Supplementary Concept] OR "empagliflozin"[All Fields]) OR ("dapagliflozin"[Supplementary Concept] OR "dapagliflozin"[All Fields] OR "dapagliflozin s"[All Fields]) OR ("canagliflozin"[MeSH Terms] OR "canagliflozin"[All Fields]) OR ("2s 3r 4r 5s 6r 2 4 chloro 3 4 ethoxybenzyl phenyl 6 methylthio tetrahydro 2h pyran 3 4 5 triol"[Supplementary Concept] OR "2s 3r 4r 5s 6r 2 4 chloro 3 4 ethoxybenzyl phenyl 6 methylthio tetrahydro 2h pyran 3 4 5 triol"[All Fields] OR "sotagliflozin"[All Fields]) OR ("ertugliflozin"[Supplementary Concept] OR "ertugliflozin"[All Fields]) OR ("bexagliflozin"[Supplementary Concept] OR "bexagliflozin"[All Fields]) OR ("ipragliflozin"[Supplementary Concept] OR "ipragliflozin"[All Fields]) OR ("6 4 ethylphenyl methyl 3 4 5 6 tetrahydro 6 hydroxymethyl spiro isobenzofuran 1 3h 2 2h pyran 3 4 5 triol"[Supplementary Concept] OR "6 4 ethylphenyl methyl 3 4 5 6 tetrahydro 6 hydroxymethyl spiro isobenzofuran 1 3h 2 2h pyran 3 4 5 triol"[All Fields] OR "tofogliflozin"[All Fields]) OR ("1 5 anhydro 1 5 4 ethoxybenzyl 2 methoxy 4 methylphenyl 1 thioglucitol"[Supplementary Concept] OR "1 5 anhydro 1 5 4 ethoxybenzyl 2 methoxy 4 methylphenyl 1 thioglucitol"[All Fields] OR "luseogliflozin"[All Fields]) OR ("licogliflozin"[Supplementary Concept] OR "licogliflozin"[All Fields]) OR ("sergliflozin"[Supplementary Concept] OR "sergliflozin"[All Fields]) OR "remogliflozin"[All Fields]))

**Supplementary Table S2. Search strategy Scopus**
(obstructive AND sleep AND apnea) AND (((glp-1ra*) OR ("glucagon like peptide 1 receptor" [mesh AND terms]) OR (glucagon-like AND peptide-1 AND receptor AND agonist*) OR (exenatide) OR (lixisenatide) OR (liraglutide) OR (dulaglutide) OR (semaglutide) OR (albiglutide) OR (efpeglenatide) OR (tirzepatide)) OR (((sodium-glucose AND co-transporter-2 AND inhibitor*) OR (sodium-glucose AND transporter 2 inhibitors[mesh AND terms]) OR (sglt-2 AND inhibitor*) OR (sodium-glucose AND cotransporter 2) OR (sglt2 AND inhibitor*) OR (sodium-glucose AND cotransporter 2 inhibitor*) OR (empagliflozin) OR (dapagliflozin) OR (canagliflozin) OR (sotagliflozin) OR (ertugliflozin) OR (bexagliflozin) OR (ipragliflozin) OR (tofogliflozin) OR (luseogliflozin) OR (licogliflozin) OR (sergliflozin) OR (remogliflozin))))

**Supplementary Table S3. Search strategy Cochrane Library**	
ID	Search
#1	obstructive sleep apnea
#2	GLP-1RA
#3	glucagon like peptide 1 receptor
#4	Glucagon-Like Peptide-1 Receptor Agonist
#5	exenatide
#6	lixisenatide
#7	liraglutide
#8	dulaglutide
#9	semaglutide
#10	albiglutide

#11	efpeglenatide
#12	tirzepatide
#13	sodium-glucose co-transporter-2 inhibitor
#14	Sodium-Glucose Transporter 2 Inhibitors
#15	SGLT-2 inhibitor
#16	sodium-glucose cotransporter 2
#17	SGLT2 inhibitor
#18	sodium-glucose cotransporter 2 inhibitor
#19	empagliflozin
#20	dapagliflozin
#21	canagliflozin
#22	sotagliflozin
#23	ertugliflozin
#24	bexagliflozin
#25	ipragliflozin
#26	tofogliflozin
#27	luseogliflozin
#28	licogliflozin
#29	sergliflozin
#30	remogliflozin
#31	#2 OR #3 OR #4 OR #5 OR #6 OR #7 OR #8 OR #9 OR #10 OR #11 OR #12
#32	#13 OR #14 OR #15 OR #16 OR #17 OR #18 OR #19 OR #20 OR #21 OR #22 OR #23 OR #24 OR #25 OR #26 OR #27 OR #28 OR #29 OR #30
#33	#1 AND (#31 OR #32)
